# Interactions between seed traits and digestive processes determine the germinability of bird-dispersed seeds

**DOI:** 10.1371/journal.pone.0195026

**Published:** 2018-04-03

**Authors:** Erik Kleyheeg, Mascha Claessens, Merel B. Soons

**Affiliations:** 1 Ecology & Biodiversity Group, Institute of Environmental Biology, Utrecht University, Utrecht, The Netherlands; 2 Department of Animal Ecology, Netherlands Institute of Ecology (NIOO-KNAW), Wageningen, The Netherlands; Sichuan University, CHINA

## Abstract

Waterbirds disperse a wide range of plant seeds via their guts, promoting biotic connectivity between isolated habitat patches. However, the intensity of digestive forces encountered by seeds, and therefore their potential to survive digestive tract passage, varies within and between waterbird species. Here, we investigate under controlled conditions how the interaction between seed traits and digestive strategies affect the germinability of seeds following waterbird-mediated dispersal. We exposed seeds of 30 wetland plant species to the main digestive processes in the dabbling duck digestive system: mechanical, chemical and intestinal digestion. These were simulated by 1) a pressure test and scarification treatment, 2) incubation in simulated gastric juice, and 3) incubation in intestinal contents of culled mallards (*Anas platyrhynchos*). We evaluated their separate and combined effects on seed germination, and identified the role of seed size and seed coat traits in resisting the digestive forces. Seeds were generally resistant to separate digestive processes, but highly sensitive to a combination. Resistance to mechanical break-down was reduced by up to 80% by chemical pre-treatment, especially for seeds with permeable coats. Scarified seeds were 12–17% more vulnerable to chemical and intestinal digestive processes than undamaged seeds. Large seeds and seeds with thin, permeable coats were particularly sensitive to chemical and intestinal digestion. These results indicate that efficient digestion of seeds requires multiple digestive processes. The gizzard, responsible for mechanical digestion, plays a key role in seed survival. Omnivorous birds, which have relatively light gizzards compared to pure herbivores or granivores, are thus most likely to disperse seeds successfully. Regardless of digestive strategy, small seeds with tough seed coats are most resistant to digestion and may be adapted to endozoochorous dispersal by waterbirds.

## Introduction

Endozoochory is the primary dispersal mode for many plant species and complementary to other dispersal modes for many others [1]. Mechanistic understanding of this functional interaction between plants and animals is necessary to uncover the extent and effectiveness of endozoochory [2,3]. In freshwater aquatic and surrounding terrestrial habitats, waterbirds, and especially dabbling ducks, disperse a wide range of aquatic, riparian and terrestrial plants [4–6]. This dispersal mechanism enables seeds to reach isolated patches in fragmented habitats and thus helps to maintain biodiversity in degraded landscapes [7]. Plants that are dispersed by waterbirds generally lack appendages or other obvious adaptations for zoochory, in contrast to, for example, plants dispersed by nonaquatic birds which often have fleshy fruits [6]. Comparative studies have shown that endozoochory is primary more frequent mechanism of waterbird-mediated dispersal than epizoochory [8,9] and diet studies have shown that dabbling ducks forage highly opportunistically on available seeds, regardless of their traits other than seed size [6,10]. Yet, large interspecific differences in gut passage survival exist, suggesting that successful endozoochory is mediated by seed size and other less conspicuous traits [11–13].

**Fig 1:**
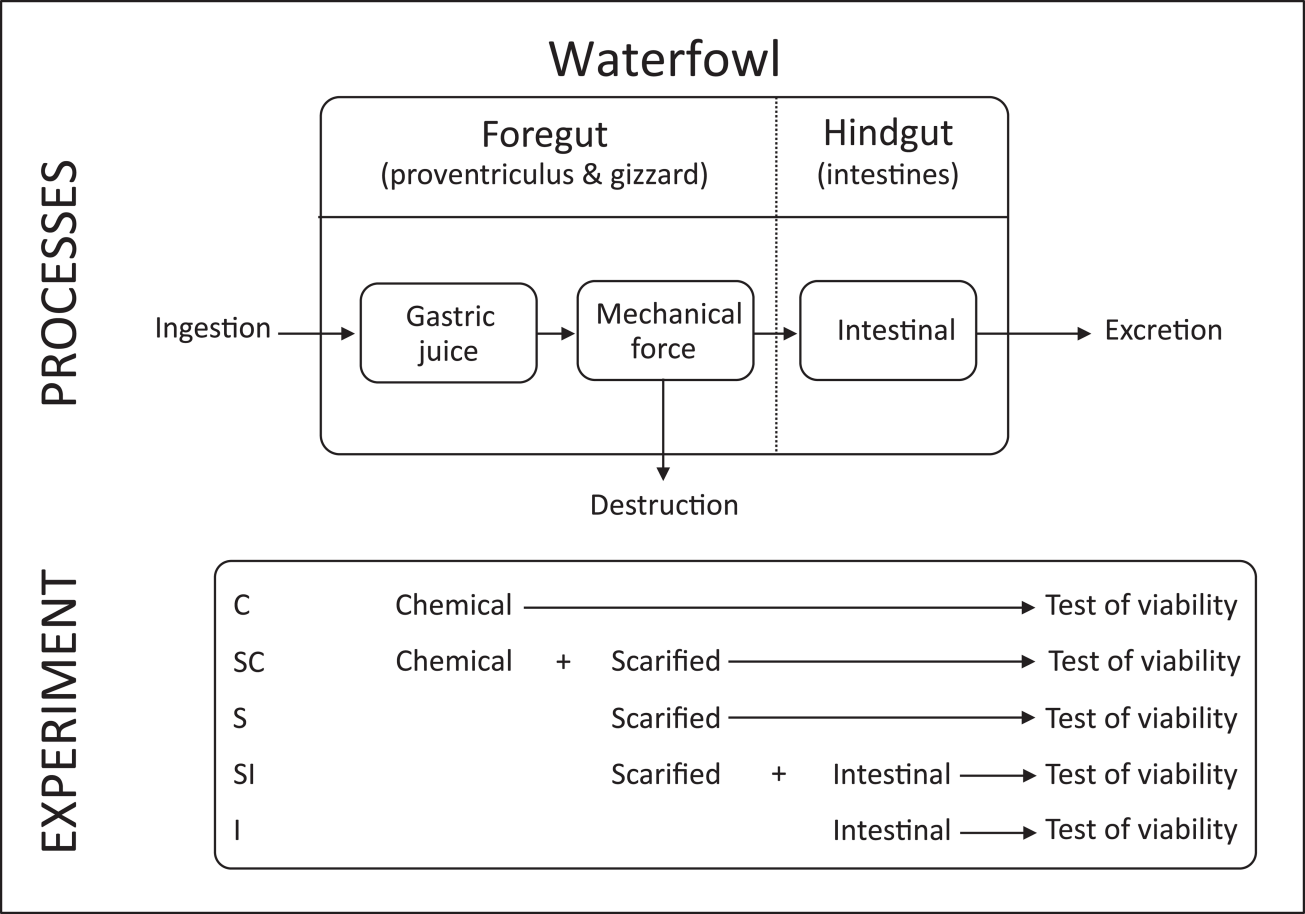
Schematic overview of digestive processes and experimental design. Top panel shows the most relevant digestive processes that seeds encounter while passing the digestive tract of a dabbling duck. Lower panel shows the simulated experimental treatments used to investigate the effect of separate digestive processes on the germination of plant seeds.

Seeds passing through the dabbling duck’s digestive tract encounter a series of consecutive sections (hereafter called *organs*) with different digestive functions (Fig 1). After retention in the crop (an elastic part of the oesophagus), ingested seeds enter the proventriculus (glandular stomach), where pepsin and hydrochloric acid are secreted, forming an acidic gastric juice [14,15]. Together with food items, this gastric juice is transported to the gizzard, where most chemical digestion takes place [14,15]. The gizzard is also responsible for mechanical breakdown of hard and large food items like seeds, using a combination of muscle strength and ingested hard particles (grit). If seeds are not destroyed in the gizzard, the mechanical and/or chemical forces encountered there may damage the seed coat (scarification), which may either reduce or stimulate germination after excretion [12,16,17]. Seeds surviving gastric digestion enter the small intestine, where enzymes are released to digest starch, fat and proteins [14,15], and the bacterial flora digests and ferments fibres and other carbohydrates [18]. Both may affect the viability of passing seeds, especially those with a damaged seed coat. In two ceca at the end of the small intestine, a considerable amount of microbial fermentation takes place [19,20] but seeds do not often enter them [21,22]. Finally, seeds reach the colon, where additional water and nutrients are extracted, and the final remains leave the bird as faeces through the cloaca.

The relative intensity of digestive processes that seeds encounter inside the digestive tract of waterbirds varies between bird species, individuals and seasons [22–25], depending on the diet to which the digestive tracts are adapted. Birds feeding mainly on invertebrates have digestive systems with smaller gizzards and rely more on chemical, and less on mechanical digestion than herbivorous species [26,27]. Omnivorous waterbirds, such as most dabbling ducks, may switch seasonally between diets that are dominated by either animal or plant components, with corresponding changes in gizzard size and intestine length [28]. Within individuals, the digestive organs are highly plastic and may respond morphologically within days to changes in dietary fibre content [23,24].

The diversity of digestive processes and variable intensity of digestive forces between and within individuals are likely to greatly affect seed gut passage survival [25,29], but very little is known about their interactions with seed traits. Most feeding experiments examining gut passage survival of ingested plant seeds treat the inside of waterbirds as a ‘black box’, where seeds enter and exit. These experiments have shown that seed traits, most importantly size (e.g. [11,12]), seed coat thickness [12,30], fibre content [11,31], and/or seed coat permeability [29], may affect gut passage survival, but it remains unclear how these results relate to the different digestive strategies of waterbirds.

In this study, we aimed to unravel the complex interactions between seed traits and digestive processes in a mechanistic way, in order to identify which seed traits may promote endozoochorous dispersal by waterbirds and evaluate which plant species are most likely to benefit from this mode of dispersal. To this purpose, we exposed seeds of plant species with a wide range of different seed traits to a series of simulated digestive processes (chemical, mechanical and intestinal), tested the effects on seed viability and time to first germination, and discussed these results in the light of survival and germination rates from earlier feeding experiments.

## Materials and methods

We performed a series of experiments under controlled conditions to test the effect of simulated digestive processes on the survival of seeds of 30 plant species belonging to 29 different genera (Table 1). The selection of species was based on their occurrence in the natural diet of dabbling ducks and their previous use in experimental feeding trials with high-quality data on gut passage survival and subsequent germination. Seeds directly collected from the plant were obtained from Cruydt-Hoeck (https://www.cruydthoeck.nl/), Jelitto Perennial Seeds (https://www.jelitto.com/) and B & T World Seeds (http://b-and-t-world-seeds.com/). We measured a range of seed traits to test their potential role in the resistance to digestive processes. We quantified effects of digestive processes either resulting in destruction of seeds or loss of their viability. We distinguished three major digestive processes: mechanical digestion, chemical digestion and intestinal digestion, and used germination trials to assess the post-treatment viability of the seeds. An overview of the experimental treatments for the viability test is shown in Fig 1.

**Table 1:** List of species names and seed traits of the plants used in this study.

Species	Length (mm)	Volume (mm^3^)	SCT (μm)	SCP	Hardness (N)
*Agrostemma githago*	3.06	10.48	66.40	0.26	49.31
*Althaea officinalis*	2.36	2.11	56.20	0.24	19.83
*Berula erecta*	1.63	0.80	136.18	0.11	9.20
*Bolboschoenus maritimus*	2.80	3.70	236.77	0.02	86.37
*Carex acuta*	2.63	0.81	59.06	0.25	10.74
*Chenopodium album*	1.31	0.67	43.79	0.04	20.66
*Comarum palustre*	1.45	0.81	80.26	0.13	3.98
*Echinochloa crus-galli*	3.11	3.28	41.19	0.07	4.05
*Eleocharis palustris*	1.53	0.65	131.43	0.03	14.85
*Epilobium hirsutum*	1.00	0.07	15.33	0.19	1.65
*Eupatorium cannabinum*	2.64	0.19	25.82	0.06	2.19
*Filipendula ulmaria*	2.81	0.74	50.76	0.18	2.96
*Hypericum tetrapterum*	0.76	0.05	23.63	0.07	0.55
*Iris pseudacorus*	7.66	153.78	350.14	0.11	253.45
*Juncus effusus*	0.50	0.01	9.90	0.18	1.44
*Lotus pedunculatus*	0.94	0.44	36.63	0.66	12.07
*Lycopus europaeus*	1.42	0.37	94.47	0.13	3.70
*Lysimachia vulgaris*	1.54	0.65	67.20	0.10	6.35
*Lythrum salicaria*	0.91	0.09	20.12	0.29	1.15
*Mentha aquatica*	0.68	0.06	30.27	0.30	0.81
*Phragmites australis*	1.36	0.13	10.26	0.28	4.59
*Polygonum aviculare*	2.80	1.03	79.96	0.11	16.15
*Polygonum pensylvanicum*	2.89	2.93	111.02	0.10	168.04
*Ranunculus sceleratus*	1.13	0.25	89.81	-0.02	2.85
*Rumex hydrolapathum*	3.24	5.06	27.87	0.23	16.94
*Sanguisorba officinalis*	3.15	2.17	33.22	0.10	4.90
*Sparganium erectum*	7.77	19.15	837.74	0.010	132.73
*Thalictrum flavum*	2.45	2.06	196.57	0.13	3.63
*Trifolium repens*	1.20	0.47	65.07	0.77	13.96
*Typha latifolia*	1.28	0.04	5.15	0.28	3.43

SCT = seed coat thickness; SCP = seed coat permeability expressed as proportional increase of seed volume after soaking for two hours in demineralized water. Seed hardness is given for untreated dry seeds. For each seed trait 20 seed per species were randomly selected and measured. Mean values per species are given.

### Seed traits

After having stored the seeds of all species in the dark at 4°C for several weeks, we initiated the experiment by measuring the following seed traits: seed volume, seed coat thickness and seed coat permeability, all of which have previously been shown to affect gut passage survival potential (e.g. [11,12,29,30]). Seed fibre content was not included as a separate trait, but is likely related to seed coat thickness and permeability [11,31]. We measured each trait in 20 randomly selected seeds per species. Specifically, we measured seed length, width and height to the nearest 0.01 mm using either a digital calliper or, in case of very small seeds, using a camera connected to a stereo microscope. Seed volume was calculated from the closest matching geometric shape (ellipsoid, pyramid, sphere, cone, cylinder or prism), using the size measures of each of the seeds. Seed coat thickness was measured using a stereo microscope with a camera and matching measurement software. Each of the seeds was thinly sliced with a razor and seed coat thickness was measured at five random locations to the nearest 0.01 mm, which was then averaged to obtain the average seed coat thickness per seed. Lastly, seed coat permeability was approximated by measuring proportional volume change after two hours of soaking in demineralized water at room temperature. We found that seed coat thickness and permeability were highly correlated. Therefore, we used the first component of a principal components analysis (PCA) on both seed coat traits as a measure of *seed coat strength*. The first component of this PCA explained 59% of the variance (eigenvalue of 1.2) and was positively correlated with seed coat thickness and negatively with seed coat permeability.

### Simulation of digestion: Mechanical

Mechanical forces in the gizzard may destroy seeds, after which germination will not occur, or scarify the seed coat, which may reduce or increase germination rates. We investigated seed resistance against breaking (hereafter *seed hardness*) by measuring for 20 seeds per species the average force needed to crack them using an Instron pressure device (Instron 5542). To simulate conditions inside the gizzard more closely, the same Instron pressure test was performed on 20 seeds per species which had been soaked in an acidic solution of pH 2.5, consisting of demineralized water and 1M hydrochloric acid, for two hours. In a separate test, we investigated the effect of seed coat damage due to scarification by effectively scarring the seed coats of 250 seeds per species with fine sand paper, or, in case of very hard seed coats that were not visibly damaged by sand paper, with single edge razor blades. Seeds were put to germinate immediately afterwards (details provided below).

### Simulation of digestion: Chemical

We studied the effect of gastric juices on seed viability by exposing both intact and scarified seeds to a solution mimicking the gastric juice of mallards *Anas platyrhynchos* [15]. We used demineralized water and 1M hydrochloric acid (HCl), with the addition of 1 mg/mL powdered pepsin (≥250 Pu/mg pepsin powder from porcine gastric mucosa, Sigma-Aldrich), to make a solution of approximately 250 Pu/mL pepsin and pH 2.5. Seeds of each species were immersed in 10 mL of simulated gastric juice (20 mL for the large *Iris pseudacorus* and *Sparganium erectum* seeds) in glass beakers in a stove for two hours (modal retention time in dabbling ducks [32]) at 42°C (mallard deep body temperature [33]). The seeds were put to germinate one to three days later and meanwhile stored in the dark at 4°C.

### Simulation of digestion: Intestinal

Intestinal digestion involves a range of enzymes and microbial organisms, making it difficult to mimic. Therefore, we investigated the effect of intestinal digestion on seed viability using the content of real mallard intestines. We collected the intestinal tracts of 10 recently shot wild mallards from a poultry dealer and extracted the intestinal matter from small intestines, ceca and colon under oxygen-deficient conditions to avoid chemical reactions with oxygen. Subsequently, 250 intact and 250 scarred seeds per species were incubated in the well-mixed intestinal matter in airtight plastic bags for two hours at 42°C. After incubation, the contents of the bags were sieved using 0.01 mm sieves, washed on filtration paper and sealed within plastic bags. These seeds were also put to germinate one to three days later and meanwhile stored in the dark at 4°C.

### Germination trials

Seeds at the end of all treatments were rinsed to simulate excretion in water and placed to germinate on moistened filtration paper in Petri dishes, sealed with Parafilm to avoid desiccation. The Petri dishes were placed in a greenhouse with an average temperature of 20°C and natural light conditions, between September and December 2014. For 2 months, germinated seeds were counted and removed twice a week. Seed germination was defined as the moment that the radicle penetrated the seed coat. The filtration paper was moistened once to twice per week. Since we were interested in the direct viability consequences of the isolated processes inside the digestive tract, and thus excluding other factors, seeds in Petri dishes with severe fungal infestation were cleaned by hand and put on clean filtration paper. As a control, 250 untreated seeds per species were rinsed and put to germinate under the same conditions.

### Data analysis

Seeds of seven of the 30 plant species hardly germinated, even in the control, and we restricted our statistical analysis to the remaining 23 species (Table 1). We used (generalized) linear mixed-effect models to test the effects of digestive treatment, seed traits, and the interaction between treatment and seed traits on 1) seed hardness, 2) seed viability, quantified as the proportion of seeds that had germinated within two months, and 3) time to first germination, quantified as the number of days before the first seed (of each species x treatment combination) germinated.

Effects on seed hardness were tested by linear mixed-effects models (LMMs) with the log-transformed amount of force required to crack the seeds (hardness) as dependent variable, treatment (soaked in acid or dry) as fixed factor, seed volume and seed coat strength as covariates and species as random factor.

To test the effects on viability, we used generalized linear mixed-effect models (GLMMs) with binomial error distribution and logit-link function. We included treatment as fixed factor (with control treatment as reference level), seed volume and seed coat strength as covariates, and the interactions seed volume x treatment and seed coat strength x treatment. We included plant species and start date (of the germination experiment) as random factors.

For germination rate, the same GLMMs were used as for viability, but with days to germination as dependent variable and a Poisson error distribution with log-link function. We optimized the models by stepwise deletion of non-significant terms from the full model and subsequently tested the effects of the individual terms using likelihood ratio tests between models with and without the term of interest. Tukey HSD post-hoc tests were used to test for differences between treatments. All calculations were performed with the lme4 package [34] and multcomp package [35] in R [36].

## Results

### Mechanical destruction

The force required to crack a seed was on average 8.6 N (range: 0.2–67 N) and positively related with seed volume (t = 5.8, p < 0.001), but unrelated with seed coat strength (t = -1.1, p = 0.26). Pre-treatment of seeds with an acid solution significantly reduced the required force by *ca*. 15% (t = 2.4, p = 0.016), with a most extreme reduction of 80% in *Althaea officinalis*. The volume-effect on the resistance against mechanical forces was not altered by acidic pre-treatment (interaction effect: t = 0.8, p = 0.45), but we found a strong role of seed coat strength in the effect of pre-treatment (interaction effect: t = -7.2, p < 0.001), indicating that seeds with thin and permeable seed coats suffered most from pre-treatment with acid.

### Treatment effects on germination

**Fig 2:**
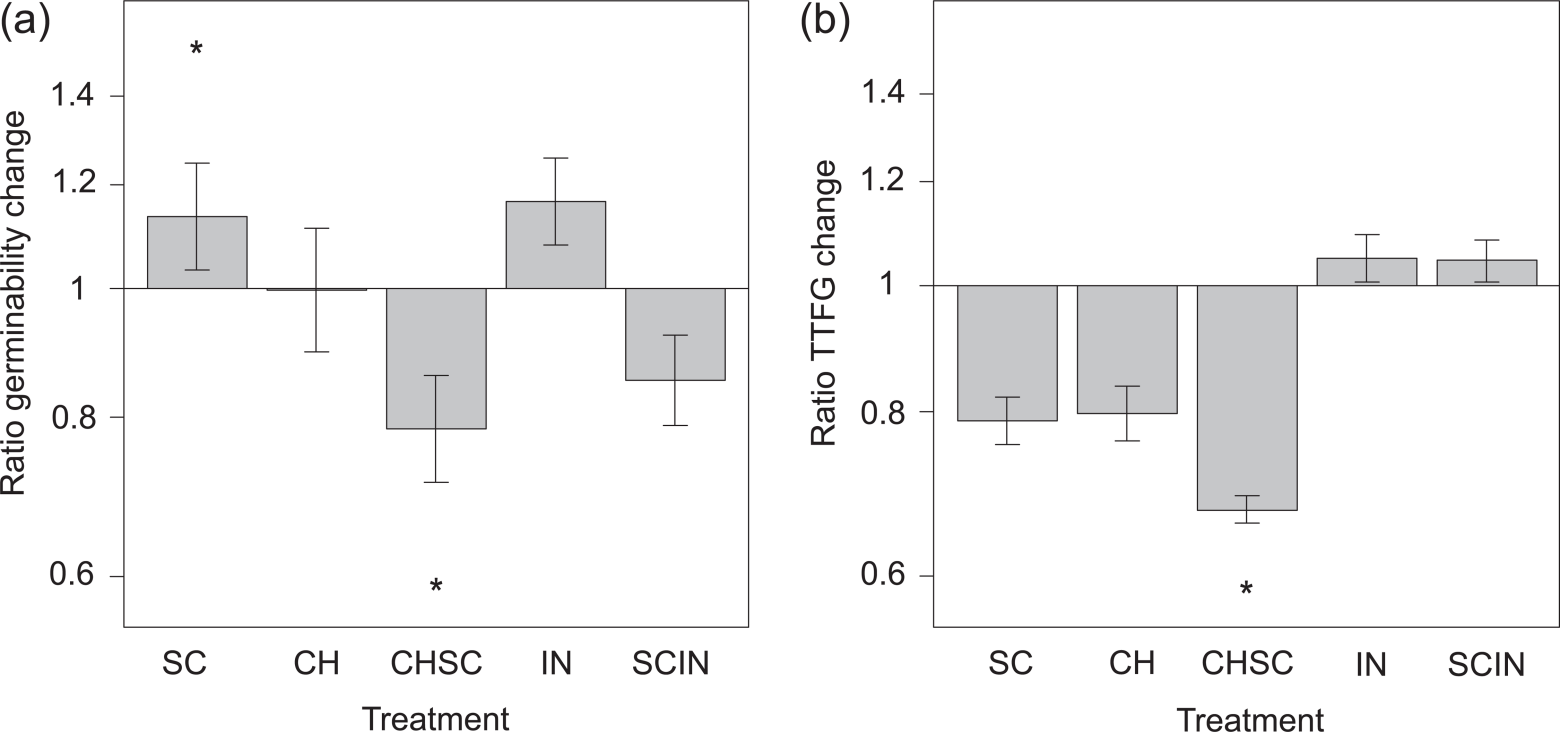
Change in viability and time to first germination change after exposure to simulated digestive processes. Change is expressed as (a) the ratio (±SE) of germination per treatment to the control treatment, and (b) the ratio (±SE) of time to first germination per treatment (TTFG) to the control treatment. Treatments are abbreviated as in Fig 1 (S = scarified, C = chemical treatment on intact seeds, SC = chemical treatment on scarified seeds, I = intestinal treatment on intact seeds, SI = intestinal treatment on scarified seeds). An asterisk indicates a significant difference from the control.

The mean proportion of seeds that germinated (viability) in the control treatment was 0.38 ± 0.07 SE (range: 0.04–0.89) and the mean time to first germination (TTFG) was 12.57 ± 1.32 SE (range: 4.2–23.2) days per species (Table 2). We found a significant effect of digestive treatment on seed viability (*χ*^2^ = 215.4, DF = 5, p < 0.001) and on TTFG (*χ*^2^ = 19.0, DF = 5, p = 0.002; Fig 2).

**Table 2:** Treatment effects on seed viability and time to first germination.

Treatment	Viability	Estimate	P-value	TTFG	Estimate	P-value
CTRL	0.38 (0.07)	-	-	12.6 (2.5)	-	-
C	0.39 (0.07)	0.16 (0.06)	0.108	9.9 (2.5)	-0.22 (0.11)	0.803
I	0.42 (0.06)	0.50 (0.37)	0.712	13.4 (2.9)	0.06 (0.14)	0.988
S	0.41 (0.06)	0.23 (0.06)	**0.002**	9.2 (2.2)	-0.19 (0.12)	0.435
SC	0.31 (0.05)	-0.52 (0.06)	**<0.001**	8.5 (2.2)	-0.39 (0.10)	**<0.001**
SI	0.34 (0.06)	0.02 (0.38)	1.000	13.2 (3.0)	-0.00 (0.14)	0.996

Effects of the separate digestive processes on mean germinability (viability) and time to first germination in days (TTFG). Standard errors are given between brackets. Significant effects (P < 0.05) are bold. Treatments are abbreviated as in Fig 1 (CTRL = untreated control, C = chemical treatment on intact seeds, I = intestinal treatment on intact seeds, S = scarification, SC = chemical treatment on scarified seeds, SI = intestinal treatment on scarified seeds).

Compared to the control treatment, the viability of intact seeds was not affected by chemical treatment (Z = 2.4, p = 0.11) or intestinal treatment (Z = 1.3, p = 0.71), but positively affected by scarification (Z = 3.7, p = 0.002). However, chemical treatment after scarification significantly reduced the viability compared to only scarified seeds (Z = -8.6, p < 0.001) as well as compared to untreated seeds (Z = -6.6, p < 0.001). Intestinal treatment did not alter the viability of scarified (Z = -0.6, p = 0.99) or untreated seeds (Z = 0.4, p = 1), but viability was significantly lower for the scarified seeds that received intestinal treatment than for intact seeds that received intestinal treatment (Z = -9.5, p < 0.001).

TTFG was not affected by scarification (Z = -1.8, p = 0.47), chemical treatment (Z = -2.1, p = 0.30), or intestinal treatment (Z = 0.4, p = 0.99). However, in scarified seeds that received chemical treatment, germination was significantly accelerated (Z = -4.0, p < 0.001). TTFG was not significantly affected by intestinal treatment, even though seeds exposed to intestinal treatment germinated relatively late (Table 2).

### Seed trait effects on germination

**Fig 3:**
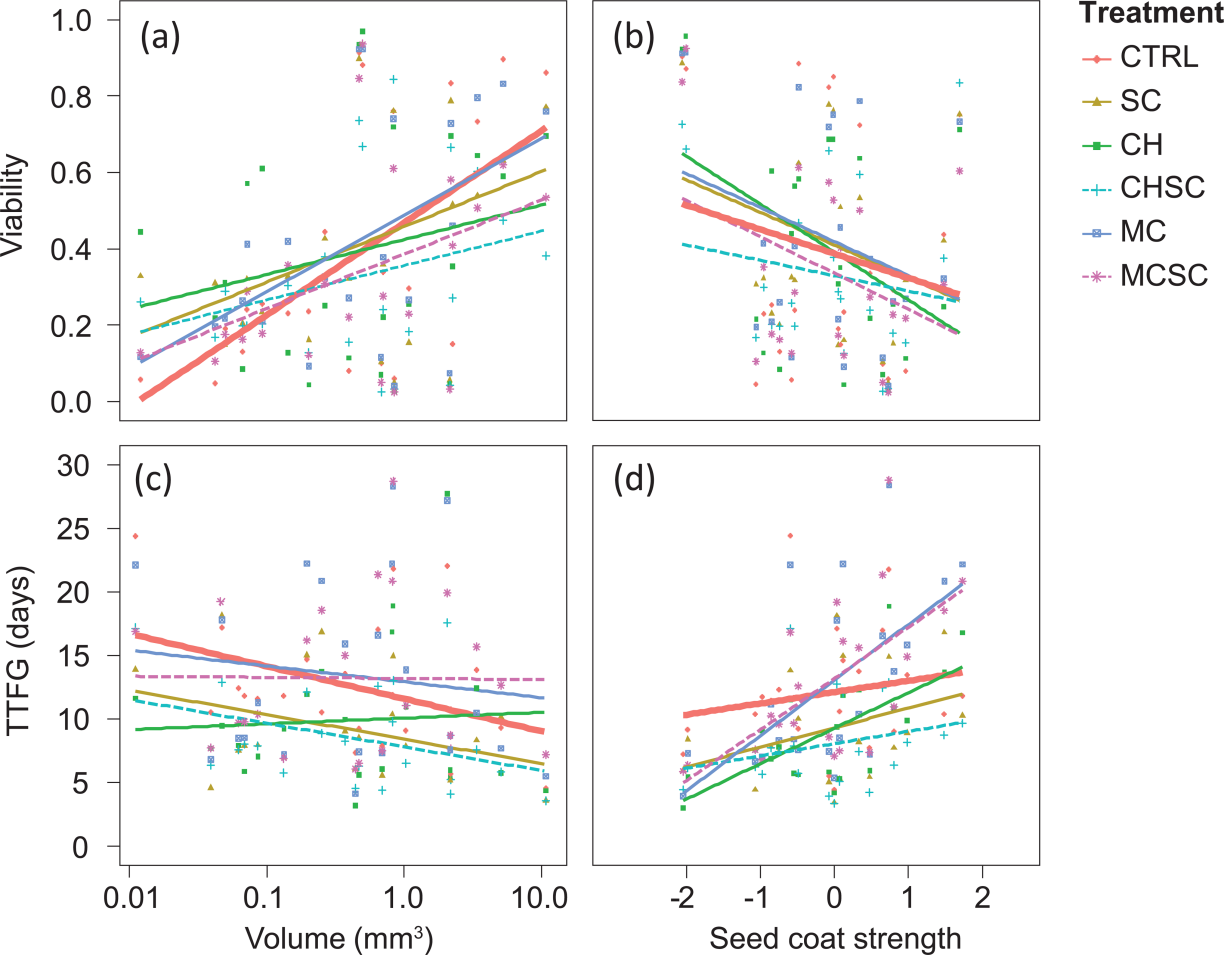
The effect of the interaction between seed traits and simulated digestion treatment on seed viability and time to first germination (TTFG). Interaction effects as represented by the slopes of the regression lines for each treatment between (a) seed volume and viability, (b) seed coat strength and viability, (c) seed volume and TTFG, and (d) seed coat strength and TTFG. Treatments are abbreviated as in Fig 1 (S = scarified, C = chemical treatment on intact seeds, SC = chemical treatment on scarified seeds, I = intestinal treatment on intact seeds, SI = intestinal treatment on scarified seeds). Thick lines represent the control treatment and dashed lines represent the combined treatments. Note that the x-axis of seed volume is log-transformed. Seed coat strength represents the first component of a PCA with seed coat thickness and permeability and is positively correlated with the former and negatively correlated with the latter.

Large seeds appeared to have higher seed viability, although this relation was not significant (χ^2^ = 2.1, DF = 1, p = 0.15; Fig 3A). Seed volume had no overall effect on TTFG (χ^2^ = 1.1, DF = 1, p = 0.28; Fig 3C). Seed coat strength, however, was negatively related with viability (χ^2^ = 6.1, DF = 1, p = 0.014) and positively related with TTFG (χ^2^ = 9.7, DF = 1, p = 0.002).

Despite the lack of an overall volume effect, viability was significantly affected by the interaction between treatment and volume (χ^2^ = 431.1, DF = 5, p < 0.001), and there was also a significant interaction between treatment and seed coat strength (χ^2^ = 192.8, DF = 5, p < 0.001; Fig 3). These interaction effects indicate that seed volume and seed coat strength both mediate the effect of digestive treatment on seed viability. Compared to the control group, all treatments significantly weakened the relation between volume and germination (Fig 3A), meaning that a potentially higher viability of larger seeds is diminished by all digestive processes. When exposed to the chemical treatment, regardless of scarification, the relation between volume and viability was weakest (Fig 3A). The relation between seed coat strength and germination was significantly stronger for seeds that received a mechanical (scarification) or chemically treated of intact seeds, compared to the control group (Fig 3B). This indicates that chemical treatment of intact seeds may act as a type of scarification, which stimulates the germination of seeds with a weak seed coat more than the germination of seeds with strong seed coats. The combined scarification and chemical treatment also more strongly affected seeds with weak seed coats, but this treatment reduced the viability.

TTFG was not significantly affected by the interactions between treatment and seed volume (χ^2^ = 6.8, DF = 5, p = 0.23, Fig 3D) or by the interaction between treatment and seed coat strength (χ^2^ = 9.1, DF = 5, p = 0.11; Fig 3C).

## Discussion

Our results demonstrate that seeds are generally well resistant against separate digestive processes, as germination rates following scarification, the chemical digestion treatment or the microbial digestion treatment were similar to germination rates of untreated control seeds. However, seeds can be strongly affected by combined digestive forces. Firstly, seeds that had received a chemical treatment were much more vulnerable to mechanical forces. Secondly, seeds that were scarified and treated with chemicals were less viable than untreated control seeds, and scarified seeds that received the intestinal treatment were less viable than intact seeds that received the same intestinal treatment. Both seed volume and seed coat strength played important roles in determining the intensity of the effect of all digestive treatments on viability, but not on TTFG. The viability of large seeds was more strongly reduced by digestive treatments than the viability of small seeds, which was most evident after chemical treatment. Seeds with weaker seed coats were more likely to germinate when scarified or treated with chemicals, but germinated less when both scarified and treated with chemicals. This suggests that abrasion of the seed coat stimulates germination, but also makes the seed more vulnerable to chemicals, which is in agreement with the overall effect of scarification and chemical treatment described above. These interaction effects indicate that seed traits are an important factor in determining gut passage survival, and that the end result of gut passage is determined by a complex interplay of seed traits and (small differences in) multiple digestive forces.

### Gastric digestion

The acidic gastric juices and strong mechanical forces in the proventriculus and gizzard potentially cause destruction, or loss of viability, of ingested seeds. In accordance with previous studies [37,38], exposure to gastric juice for two hours did not affect intact seeds. It did, however, significantly increase their vulnerability to mechanical destruction, especially for seeds with thin and permeable seed coats. Hence, even though seed coat strength at first did not seem to affect resistance to mechanical pressure, it turned out to be a crucial seed trait when the seeds had been pre-treated with acid. In either case, the cracking of larger seeds required more force. In the first instance, this appears to contradict the results of previous feeding trial studies that show that larger seeds are more often destroyed than smaller seeds [11–13]. However, larger seeds in reality face a higher probability of physical damage by grit due to 1) their larger contact area, which makes them more likely to experience damage by grit (smaller seeds may ‘escape’ mechanical forces in the cavities between grit particles), and 2) their longer retention in the gizzard (E.K. unpublished data). The seed size effect has a clear parallel in mammalian dispersers, where small and rounded seeds are less likely damaged in the mastication process and therefore more likely to survive endozoochory [39,40].

If not destroyed, seeds could still be scarified in the gizzard. By itself, scarification increased the germination of seeds, probably by breaking seed dormancy in a number of species, thereby encouraging seeds to germinate [37,41]. However, subsequent exposure to gastric juice severely reduces seed viability, also compared to seeds with undamaged seed coats. This suggests that the gizzard is a limiting factor for dispersal for most seed species. Therefore, seed species with traits that prevent scarification in the gizzard in the first place (i.e. small size and a very strong seed coat), have the highest probability of surviving gut passage.

### Intestinal digestion

Inside the intestines, enzymes and the microbial flora take another role in the digestion of food [14,15], but our simulation of intestinal digestion did not cause significant changes in the viability of intact or scarified seeds compared to untreated seeds. However, intact seeds treated with intestinal matter did show higher viability than scarified seeds that had the same treatment. During the germination experiment, we observed fungal infestation of scarified seeds. Under natural circumstances, seeds are likely to be excreted into water after digestion [29], which we simulated by rinsing the seeds after the microbial treatment. While intact seeds are supposedly washed clean easily, scarred seeds may contain leftover faecal matter within the cracked seed coat, facilitating fungal growth, which may affect viability [42]. This could explain the lower germination of scarified seeds treated with intestinal matter.

### Effects of seed traits

In general, larger seeds appeared more viable and germinated earlier, although both relations were not significant. Yet, all digestive treatments reduced the viability of large seeds more than that of small seeds. Even the most effective treatment (chemical + scarified), however, still yielded a higher germinability of larger seeds. This suggests that our treatments were relatively mild for large species in comparison to real digestive forces operating in dabbling ducks, as germination following mallard gut passage was significantly reduced in larger as opposed to smaller seeds for 23 similar species (of which 12 were the same as in this study) in an earlier feeding experiment by Soons et al. [12]. The longer retention in the digestive tract [29] likely explains part of this greater loss of viability in large seeds measured after gut passage. Our study demonstrates that even when retention time is kept constant, the viability of large seeds is more strongly reduced by digestive processes than that of small seeds. The large contact area of large seeds could make them more sensitive to digestive processes. Chemical treatment had a stronger effect on the relation between size and viability than intestinal treatment, emphasizing the importance of the gastric function for seed digestion.

Seed coats generally hamper germination (e.g. [41]), so that damaging the seed coat may stimulate germination. The negative relation between seed coat strength and germination is amplified by chemical treatment and scarification separately, which could indicate that weak seed coats are more easily broken by these forces than thick seed coats, thereby especially stimulating germination of seeds with weak seed coats. Together, chemical treatment and scarification however lowered the seed coat effect on viability, indicating that when the protective seed coat is scarified, seeds become more vulnerable to chemical treatment, diminishing the stimulating effect of a damaged seed coat on germination.

Overall, TTFG was rather insensitive to digestive processes and only scarification combined with chemical treatment resulted in a significant acceleration of *ca*. four days (33%) compared to the control. Accelerated germination can be beneficial as it reduces the time for fungal infestation or seed predation, but based on this study it is difficult to predict if passage through the entire digestive tract will indeed lead to an acceleration of germination.

### Comparison with feeding trials

**Fig 4:**
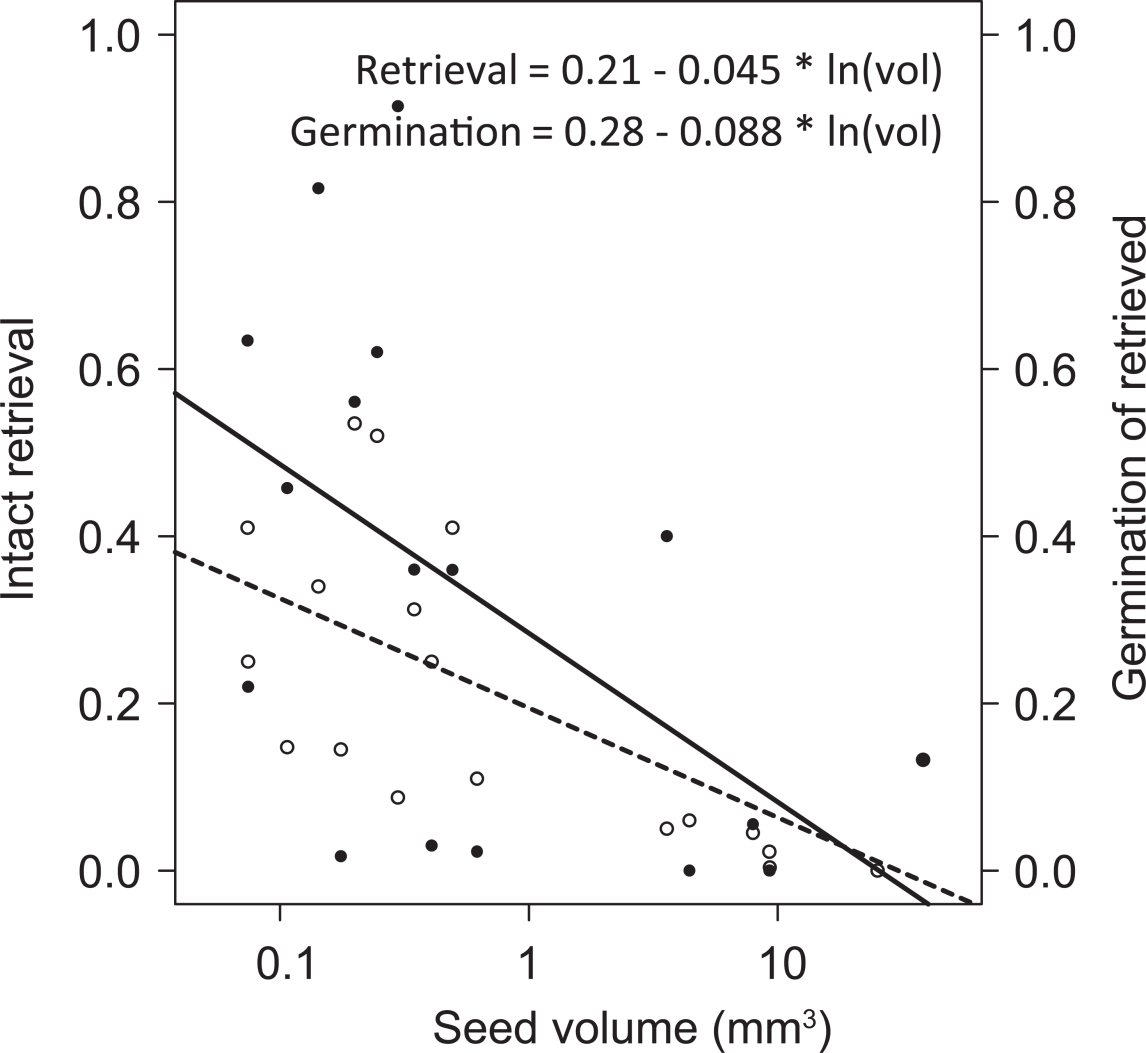
Relation between seed volume and gut passage survival. The dotted line (open circles) shows the negative relation between seed volume and gut passage as a proportion of retrieved seeds, and the solid line (closed circles) shows the relation between seed volume and germination (viability) after gut passage. Based on data from the feeding experiment performed by Soons et al. (2008) [12].

To place the results of this study in the context of actual digestion by waterbirds, and to point out which elements of the ‘black box’ are most important for gut passage survival, we compared our results with results from experimental feeding trials. Most importantly, feeding trials unequivocally demonstrate that destruction of seeds in the gizzard, rather than scarification, chemical or intestinal treatments, is limiting for viable gut passage. Especially large seeds have low probabilities of passing the gizzard undamaged in the first place, despite the larger force required to break them [11–13,43,44]. After losses due to breakage, the viability of defecated intact seeds depends on effects of combined scarification and chemical treatment, mediated by seed volume and seed coat strength. Again, large seeds suffer the most, as shown by the strong negative relation between seed size and viability of retrieved seeds in a study by Soons et al. [12] (Fig 4). However, also seeds with weaker seed coats suffer greatly from the combined treatment. The latter may explain the remarkably low survival of the smallest seeds used in Kleyheeg et al. [43], which indeed had a very permeable seed coat, and this seems to be the second-most important determinant of intact gut passage. Wongsriphuek et al. [31] showed that seeds with high fibre content (indicating a stronger seed coat) had a higher probability of intact gut passage and a lower germination, supporting the seed coat effects found in this study.

In general, the germinability of treated versus control seeds was relatively high compared to feeding trial studies (e.g. [12,43]). We have not tested the cumulative effect of the mechanical, chemical and intestinal treatments, which would more closely resemble the passage through the entire digestive tract and likely reduce general viability. Also, we did not account for all factors potentially contributing to viability loss. In particular, we did not specifically test the effect of exposure to relatively high body temperatures. In an experimental digestive tract simulation of mammalian herbivores, germination was generally inhibited after incubation at 38°C for 24–72 hours [45]. In our study, incubation at 42°C for two hours in combination with intestinal treatment did not reduce germinability compared to untreated seeds, but in studies of long-distance dispersal, the prolonged exposure to high temperatures should be taken into account. Moreover, this temperature effect is likely an additional factor mediating viability consequences of longer retention times of large seeds.

Additional gut simulation experiments in combination with feeding trials will be useful to further disentangle the effect of variability in digestive tract function within and between potential dispersers. In particular, testing effects of exposure time to digestive processes is necessary to assess the potential for long-distance dispersal, while exposing seeds to different acidity levels and enzymes will help understand how seeds are affected by digestion in animals with different digestive strategies.

### Implications for seed dispersal

Our results, detailing the mechanisms underlying the effects of seed traits on waterbird gut passage survival, allow better estimation of survival and dispersal rates of seeds under natural conditions, and estimation of the effectiveness of dispersal by specific seed-vector pairs. Many duck and other waterbird species seasonally change their diets. Their guts have the ability to adapt rapidly to changes in the quality and quantity of the food: a seed-based (high fibre) diet in autumn and winter results in a larger gizzard and longer intestines in comparison to an animal-based (low-fibre) diet in spring and summer [23,24,46]. This may have major consequences for seed dispersal [25,29]. In late autumn and winter, the larger gizzard is likely to increase seed destruction, but may also cause more scarification of the seed coat, decreasing viability if the scarified seed is exposed to gastric juices. Seeds consumed by waterbirds on a primarily animal-based diet are less likely to be scarified due to the smaller gizzard, and may thus have a higher viability after gut passage. Retention time also decreases when birds are on a low-fibre diet, which may further increase seed viability after gut passage [12,25]. Thus, seeds ingested by dabbling ducks in late summer and early autumn have a high probability of intact and viable gut passage, and hence dispersal, independent of seed traits. As autumn progresses and waterbird switch to a more fibre-rich diet, seed traits become increasingly important. In late winter, only small seeds with tough seed coats maintain a high probability to be successfully dispersed by dabbling ducks, as these are best adapted to survive gut passage under all conditions. Seed size and seed coat hardness are also implicated in the survival of digestive tract passage in mammals [39,40,47], hinting at universal traits for adaptation of plants to endozoochorous dispersal.
